# Malnutrition-related diabetes mellitus: severe food insecurity on international agendas and implications for public health in Brazil

**DOI:** 10.1590/0102-311XEN097125

**Published:** 2025-10-03

**Authors:** Daniel Ferreira da Silva, Dirce Maria Lobo Marchioni

**Affiliations:** 1 Faculdade de Saúde Pública, Universidade de São Paulo, São Paulo, Brasil.

The role of diet as a central determinant for the occurrence of chronic noncommunicable diseases, especially in endocrine-metabolic conditions related to obesity ^1^ and Western dietary patterns ^2^, is well established ^3^. However, the prevailing logic that exclusively associates excess calories with illness ignores the other side of a complex public health problem: the harmful effects of chronic exposure to severe food insecurity ^4^, recently highlighted by the recognition of malnutrition-related diabetes mellitus (MRDM) by the International Diabetes Federation (IDF) ^5^.

This presentation of the disease has gained prominence, mainly through studies led by Meredith Hawkins, who compared the clinical and metabolic profiles of individuals susceptible to a diagnosis of MRDM with those of patients with type 1 (DM1) and type 2 (DM2) diabetes mellitus ^6^. It was demonstrated that these individuals, who were negative for autoantibodies as well as monogenic and lipoatrophic forms of the disease, had lower pancreatic insulin reserves compared to those with DM2, but higher levels than those found in DM1. Moreover, the subjects had preserved insulin sensitivity, with lower endogenous production and greater peripheral glucose uptake compared to those with DM2, revealing a fundamentally distinct clinical phenotype ^7^.

Severe food insecurity is the recurring experience of hunger resulting from the violation of the human right to adequate food and is a central condition for understanding MRDM. According to the Food and Agriculture Organization of the United Nations (FAO), families in situations of severe food insecurity are likely to go without food, and their members may go a day or more without eating during the reference period ^8^. In Brazil, this condition is measured primarily through the *Brazilian Food Insecurity Scale* (EBIA, acronym in Portuguese), which classifies households as having mild, moderate, or severe food insecurity based on the experiences of family members ^9^.

During the 2025 World Diabetes Congress in Bangkok, Thailand, in addition to officially recognizing the disease as type 5 diabetes mellitus (DM5), the IDF announced the creation of a working group for defining diagnostic criteria, developing therapeutic guidelines, and establishing a global case registry ^5^. The initiative also discussed the development of educational modules for healthcare providers and the promotion of international research collaborations ^5^. This decision reignited debate in the scientific community on this form of presentation of diabetes, described nearly 70 years ago, but still absent from official diagnostic classifications.

MRDM was first described in 1955 by British physician Hugh-Jones, using data from Jamaica ^10^. Other descriptions followed, mainly in countries of sub-Saharan Africa and Asia ^11^ ^,^ ^12^ ^,^ ^13^ ^,^ ^14^ ^,^ ^15^, with prevalence data showing the DM5 accounted for approximately 23% of all cases of diabetes in India ^16^. These patients were generally young and thin, with a low socioeconomic status and a history of chronic malnutrition, manifesting diabetes with clinical characteristics distinct from the classic profiles of the disease ^17^. In 1985, the World Health Organization (WHO) ^18^ officially recognized this form of presentation, calling it “malnutrition-related diabetes mellitus”, which initially fostered proposals for diagnostic criteria ^15^. However, the classification was removed from official documents years later due to a lack of studies supporting the differentiation ^19^.

There is consistent evidence, however, that populations with severe food insecurity, especially at the beginning of life, have a greater risk of developing metabolic and cardiovascular conditions in adulthood ^20^ ^,^ ^21^. This relationship was initially proposed in the pioneering work of David Barker et al. ^22^, who described the association between low birth weight, which is an indicator of poor nutrition during pregnancy, and heart disease in adulthood. The group led by Barker subsequently expanded on these observations, identifying associations between low birth weight and other conditions, such as glucose intolerance, DM2, systemic arterial hypertension, and dyslipidemia ^23^ ^,^ ^24^ ^,^ ^25^.

This scenario may have even more profound implications in regions historically marked by social inequalities, such as Latin America, where obesity and nutritional deficiencies often coexist in the same person, family, or community, characterizing the double burden of malnutrition ^26^. In Brazil, for instance, a cross-sectional analysis of the baseline of the *Brazilian Longitudinal Study of Aging* (ELSI-Brazil, acronym in Portuguese) showed that one-quarter of the population 50 years of age or older experienced hunger in childhood, especially those who resided in the North and Northeast regions of the country ^27^. Such individuals were 20% more likely to develop diabetes in adulthood ^27^.

The delayed recognition of DM5 may reflect weaknesses in the global commitment to ensure the human right to adequate food by neglecting the fact that hunger can leave profound biological scars. Moreover, one should consider that the clinical phenotype of malnutrition-related diabetes resembles that of DM1 in many respects, affecting young and lean individuals, who are often treated with full insulin therapy ^6^. Thus, many of these cases may be misclassified in populations historically affected by severe food insecurity ^6^. This gives rise to an inevitable question: how many cases of diabetes diagnosed in low- and middle-income countries are clinical manifestations of chronic malnutrition?

Post-COVID-19 pandemic estimates revealed that more than 33 million people faced hunger in Brazil, with 58.7% of families experiencing some degree of hunger and at least 15.5% experiencing severe hunger ^28^. This context of extreme hunger is linked to another alarming fact: Brazil ranks sixth in the world in terms of the number of cases of diabetes, with estimates from the *Diabetes Atlas* ^29^ that 16.6 million people lived with the disease in 2024 and that this number could reach 24 million by 2050. The IDF also estimates that between 20 and 25 million people throughout the world are affected by DM5 ^5^. Given this scenario, it is plausible that Brazil’s historical and persistent experience with hunger contributes to the increase in statistics on diabetes through cases directly related to malnutrition. Future studies, such as ongoing birth cohorts in Brazil ^30^, will play a fundamental role in clarifying the impact of severe food insecurity on the prevalence of this form of disease presentation in the country.

Although a complete clinical history should include the patient’s social background and life experiences, diagnoses of chronic noncommunicable diseases are based primarily on clinical and laboratory findings, with little or no consideration of socioeconomic factors ^31^. The recognition of MRDM, therefore, constitutes a paradigm shift by proposing that the formulation of the diagnosis should consider social dimensions as an inseparable part of the illness process ^6^. 

In Brazil, the use of data on chronic exposure to hunger for the generation of relevant statistics could include information on low birth weight ^32^, self-reported severe food insecurity in childhood, or short stature in adulthood as a proxy for childhood malnutrition ^33^. Moreover, households experiencing food insecurity could be identified early through *Screening Households at Risk of Food Insecurity* (TRIA, acronym in Portuguese) ^34^, which is a two-item method recently incorporated into the Brazilian Health Information System for Primary Care (SISAB, acronym in Portuguese) to detect situations of food vulnerability in an agile, standardized manner across the services of the Brazilian Unified National Health System (SUS, acronym in Portuguese) ^35^.

The renewed discussion on MRDM also paves the way for the reformulation of public health policies aimed at the protection of pregnancy and early childhood, as the determinants of this form of diabetes often begin in utero, when the mother’s exposure to food insecurity and malnutrition can compromise fetal development and biologically program greater metabolic vulnerability throughout life ^6^ ^,^ ^20^ ^,^ ^21^. Therefore, strengthening policies that ensure food and nutritional security during pregnancy and in the first years of life is essential to preventing both malnutrition and its long-term repercussions, such as the emergence of atypical forms of diabetes. Interventions in this critical period of life have the potential to break intergenerational cycles of poverty, hunger, and illness, contributing to health equity and addressing the structural social inequalities that mark the Brazilian scenario.

The initiative of the IDF to officially recognize MRDM and create an international working group to define diagnostic and therapeutic criteria constitutes a significant milestone in understanding the multiple determinants of diabetes in different socioeconomic contexts. It also constitutes an important shift in how global public health faces social determinants of endocrine and metabolic diseases.

The inclusion of this topic on global agendas drives a broader understanding of the pathophysiological mechanisms associated with the effects of prolonged hunger, underscoring the need to produce knowledge that is more sensitive to the situations experienced by populations historically marked by deep social inequalities. While there is no consensus on the diagnostic criteria or therapeutic approaches, the inclusion of this topic on the agenda of an international organization such as the IDF paves the way for new studies and revisions, turning greater attention to the disease, especially in low- and middle-income countries, where severe food insecurity and malnutrition are persistent challenges.
