# Supplementary material for: Malnutrition-related diabetes mellitus: severe food insecurity on international agendas and implications for public health in Brazil
Source: Cad Saude Publica. 2025 Oct 3;41(9):e00097125. doi: 10.1590/0102-311XEN097125 (PMC12494382; doi:10.1590/0102-311XEN097125)
Supplement: Translation

# Translation: Diabetes mellitus relacionado à desnutrição: a insegurança alimentar grave nas agendas internacionais e suas implicações para a saúde pública no Brasil

**DOI:** 10.1590/0102-311XPT097125

**Authors:** Daniel Ferreira da Silva, Dirce Maria Lobo Marchioni

**Affiliations:** 1 Faculdade de Saúde Pública, Universidade de São Paulo, São Paulo, Brasil.

O papel da alimentação como determinante central para a ocorrência de doenças crônicas não transmissíveis (DCNT), especialmente, em condições endocrinometabólicas relacionadas à obesidade ^1^, e a padrões alimentares ocidentalizados ^2^, está bem estabelecido ^3^. No entanto, a lógica predominante que associa exclusivamente o excesso de calorias ao adoecimento ignora a outra face de um problema de saúde pública complexo: os efeitos deletérios da exposição crônica à insegurança alimentar grave ^4^, recentemente evidenciados pelo reconhecimento do diabetes mellitus relacionado à desnutrição (DMRD) pela Federação Internacional de Diabetes (IDF, acrônimo em inglês) ^5^.

Essa forma de apresentação da doença tem ganhado destaque, principalmente, pelos estudos liderados por Meredith Hawkins, que comparou o perfil clínico e metabólico de indivíduos suscetíveis ao diagnóstico de DMRD a pacientes com diabetes mellitus tipo 1 (DM1) e tipo 2 (DM2) ^6^. Foi demonstrado que esses indivíduos, negativos para autoanticorpos e para formas monogênicas e lipoatróficas da doença, possuíam menor reserva pancreática de insulina em relação ao DM2, mas com níveis superiores aos observados no DM1. Além disso, apresentaram sensibilidade à insulina preservada, com menor produção endógena e maior captação periférica de glicose em comparação ao DM2, evidenciando um fenótipo clínico fundamentalmente distinto ^7^.

A insegurança alimentar grave, condição central para a compreensão do DMRD, refere-se à experiência recorrente de fome decorrente da violação do direito humano à alimentação adequada. De acordo com a Organização das Nações Unidas para a Alimentação e a Agricultura (FAO, acrônimo em inglês), famílias nessa situação provavelmente ficarão sem comida e seus membros poderão passar um dia ou mais sem se alimentar durante o período de referência ^8^. No Brasil, a mensuração dessa condição é realizada, principalmente, por meio da *Escala Brasileira de Insegurança Alimentar* (EBIA), que permite classificar os domicílios em insegurança alimentar leve, moderada ou grave, com base nas experiências vividas pelos membros da família ^9^.

Durante o Congresso Mundial de Diabetes de 2025, realizado em Bangkok, Tailândia, além de reconhecer oficialmente a doença, denominando-a como diabetes mellitus tipo 5 (DM5), a IDF anunciou a criação de um grupo de trabalho responsável por definir critérios diagnósticos, elaborar diretrizes terapêuticas e estabelecer um registro global de casos ^5^. A iniciativa discutiu, ainda, o desenvolvimento de módulos educacionais para profissionais de saúde e a promoção de colaborações internacionais em pesquisa ^5^. Essa decisão reacendeu o debate na comunidade científica sobre essa forma de apresentação do diabetes, descrita há quase 70 anos, mas ainda ausente nas classificações diagnósticas oficiais.

A primeira descrição do DMRD foi realizada em 1955, pelo médico britânico Hugh-Jones, com dados oriundos da Jamaica ^10^. Em seguida, outras descrições foram realizadas, principalmente em países da África Subsaariana e Ásia ^11^ ^,^ ^12^ ^,^ ^13^ ^,^ ^14^ ^,^ ^15^, com dados de prevalência na Índia de aproximadamente 23% dos casos de diabetes ^16^. Esses pacientes, em geral, eram jovens, magros, de baixo *status* socioeconômico e com histórico de desnutrição crônica, manifestando um diabetes com características clínicas distintas dos perfis clássicos da doença ^17^. Em 1985, a Organização Mundial da Saúde (OMS) ^18^ chegou a reconhecer oficialmente essa forma de apresentação, denominando-a *malnutrition-related diabetes mellitus* (diabetes mellitus relacionada à desnutrição), o que fomentou, inicialmente, propostas de critérios diagnósticos ^15^. Entretanto, anos mais tarde, a classificação foi retirada dos documentos oficiais por alegação de escassez de estudos que sustentassem a diferenciação ^19^.

Existem evidências consistentes, no entanto, de que populações em situação de insegurança alimentar grave, especialmente durante o início da vida, apresentam maior risco de desenvolver condições metabólicas e cardiovasculares na idade adulta ^20^ ^,^ ^21^. Essa relação foi proposta, inicialmente, pelo trabalho pioneiro de David Barker et al. ^22^, ao descrever a associação entre baixo peso ao nascer - um indicador de má nutrição durante a gestação - e doença cardíaca na vida adulta. Posteriormente, o grupo liderado por Barker ampliou essas observações, identificando associações entre baixo peso ao nascer e outras condições como intolerância à glicose, DM2, hipertensão arterial sistêmica e dislipidemias ^23^ ^,^ ^24^ ^,^ ^25^. 

Esse cenário pode ter implicações ainda mais profundas em regiões historicamente marcadas por desigualdades sociais, como na América Latina, onde a obesidade e as carências nutricionais coexistem, muitas vezes, em uma mesma pessoa, família ou comunidade, caracterizando a dupla carga de má nutrição ^26^. No Brasil, por exemplo, uma análise transversal da linha de base do *Estudo Longitudinal da Saúde dos Idosos Brasileiros* (ELSI-Brasil) evidenciou que um quarto da população com 50 anos ou mais de idade havia passado fome na infância, sobretudo nas regiões Norte e Nordeste ^27^. Esses indivíduos apresentaram 20% mais chances de desenvolverem diabetes na idade adulta ^27^.

O reconhecimento tardio do DM5 pode refletir fragilidades no compromisso global com a garantia do direito humano à alimentação adequada, ao negligenciar que a fome pode deixar marcas biológicas profundas. Além disso, há de se considerar que o fenótipo clínico do DMRD se assemelha, em muitos aspectos, ao DM1, afetando indivíduos jovens e magros, frequentemente tratados com insulinização plena ^6^. Assim, é possível que muitos desses casos possam ser erroneamente classificados em populações historicamente afetadas pela insegurança alimentar grave ^6^. Diante desse cenário, surge uma pergunta inevitável: quantos dos casos de diabetes diagnosticados em países de baixa e média renda não seriam manifestações clínicas da desnutrição crônica? 

Estimativas pós-pandemia de COVID-19 revelaram que mais de 33 milhões de pessoas enfrentavam a fome no Brasil, com 58,7% das famílias em algum grau de insegurança alimentar e pelo menos 15,5% em situação de insegurança alimentar grave ^28^. Esse contexto de insegurança alimentar extrema dialoga com outro dado alarmante: o Brasil é o sexto país do mundo com o maior número de casos da doença, com estimativas do *Atlas do Diabetes* ^29^ de que 16,6 milhões de pessoas conviviam com a doença em 2024 e que esse número pode chegar a 24 milhões até 2050. A IDF também estima que entre 20 e 25 milhões de pessoas no mundo são afetadas pelo DM5 ^5^. Diante desse cenário, é plausível considerar que a experiência histórica e persistente da fome no país contribua para o aumento das estatísticas de diabetes, por meio do surgimento de casos diretamente relacionados à desnutrição. Pesquisas futuras, como as coortes de nascimento em andamento no Brasil ^30^, terão um papel fundamental em esclarecer o impacto da insegurança alimentar grave na prevalência dessa forma de apresentação da doença no país.

Cabe destacar que, embora uma anamnese clínica completa deva incluir antecedentes sociais e experiências de vida do paciente, os diagnósticos de DCNT são baseados majoritariamente em parâmetros clínicos e laboratoriais, com pouca ou nenhuma incorporação de aspectos socioeconômicos ^31^. O reconhecimento do DMRD representa, portanto, uma quebra de paradigma ao propor que a formulação diagnóstica considere dimensões sociais como parte indissociável do processo de adoecimento ^6^. 

No Brasil, a operacionalização dos dados de exposição crônica à fome para a geração de estatísticas de interesse poderá ser realizada com informações sobre baixo peso ao nascer ^32^, autorrelato de insegurança alimentar grave na infância ou baixa estatura na vida adulta como *proxy* de desnutrição infantil ^33^. Além disso, domicílios em situação de insegurança alimentar poderão ser identificados precocemente por meio da *Triagem para Risco de Insegurança Alimentar* (TRIA) ^34^, um *screener*, composto de dois itens, recentemente incorporado no Sistema de Informação em Saúde para a Atenção Básica (SISAB), com o objetivo de detectar situações de vulnerabilidade alimentar de forma ágil e padronizada nos serviços do Sistema Único de Saúde (SUS) ^35^.

A retomada da discussão sobre o DMRD também abre espaço para a reformulação de políticas públicas de saúde voltadas à proteção da gestação e da primeira infância. Isso porque os determinantes dessa forma de diabetes frequentemente se iniciam na vida intrauterina, quando a exposição da gestante à insegurança alimentar e à má nutrição pode comprometer o desenvolvimento fetal e programar, biologicamente, maior vulnerabilidade metabólica ao longo da vida ^6^ ^,^ ^20^ ^,^ ^21^. Portanto, fortalecer políticas que garantam segurança alimentar e nutricional no período gestacional e nos primeiros anos de vida é essencial para prevenir não apenas a desnutrição, mas também suas repercussões de longo prazo, como o surgimento de formas atípicas de diabetes. Intervenções nesse período crítico do curso da vida têm potencial de romper ciclos intergeracionais de pobreza, fome e adoecimento, contribuindo para a equidade em saúde e para o enfrentamento das desigualdades sociais estruturais que marcam o cenário brasileiro. 

A iniciativa da IDF de reconhecer oficialmente o DMRD, e criar um grupo de trabalho internacional para definir critérios diagnósticos e terapêuticos, representa um marco significativo na compreensão dos múltiplos determinantes do diabetes em diferentes contextos socioeconômicos, além de representar uma inflexão importante na forma como a saúde pública global encara os determinantes sociais de doenças endocrinometabólicas. 

A inclusão desse tema nas agendas globais impulsiona a ampliação da compreensão dos mecanismos fisiopatológicos associados aos efeitos da fome prolongada, reforçando a necessidade de se produzir conhecimento mais sensível às realidades vividas por populações marcadas historicamente por profundas desigualdades sociais. Ainda não há consenso quanto a critérios diagnósticos ou abordagens terapêuticas. No entanto, a inclusão do tema na pauta de uma organização internacional como a IDF abre caminho para novas pesquisas e revisões, possibilitando maior atenção à doença, especialmente em países de baixa e média renda, onde a insegurança alimentar grave e a desnutrição são desafios persistentes.
